# Endovascular Intervention for Aortic Dissection Is “Ascending”

**DOI:** 10.3390/ijerph20054094

**Published:** 2023-02-24

**Authors:** Antonio Rizza, Francesco Negro, Tim J. Mandigers, Cataldo Palmieri, Sergio Berti, Santi Trimarchi

**Affiliations:** 1 Cardiology Unit, Ospedale del Cuore, Fondazione Toscana “G. Monasterio”, 54100 Massa, Italy; 2 Cardiology Division, Pisa University Hospital, 56124 Pisa, Italy; 3 Department of Vascular Surgery, Fondazione IRCCS Cà Granda Ospedale Maggiore Policlinico, 20122 Milan, Italy; 4 Clinical and Community Sciences Department, Università degli Studi di Milano, 20122 Milan, Italy

**Keywords:** atherosclerosis, aortic disease, aortic dissection, aortic aneurysm, percutaneous intervention

## Abstract

Ascending aorta diseases represent an important cause of mortality worldwide. Notably, acute and chronic thoracic aorta pathologies have increased during the last years, but medical therapy does not seem to influence their natural history. Currently, although open surgery is the first choice of treatment, many patients are still rejected or have poor outcomes. In this scenario, endovascular treatment is raised as a valuable option. In this review we describe the limitations of conventional surgery and the state-of-art of endovascular ascending aorta repair.

## 1. Introduction

Mortality from aortic aneurysm in Western countries during the twentieth century has decreased. Prevalence of abdominal aortic aneurysm (AAA) in 65-year-old men is about 4% according to ultrasound screening studies [1], rising to 5% in the population aged 65–74 years [2].

A different trend exists in patients with thoracic aortic aneurysm (TAA): from 1987 through 2002, the incidence of thoracic aorta dissection increased significantly in both sexes, but it appears more pronounced in men. An increase of 52% and 28% was observed in males and females, respectively [3]. Moreover, the median age at diagnosis significantly decreased, from 73 years to 71 years.

**Figure 1:**
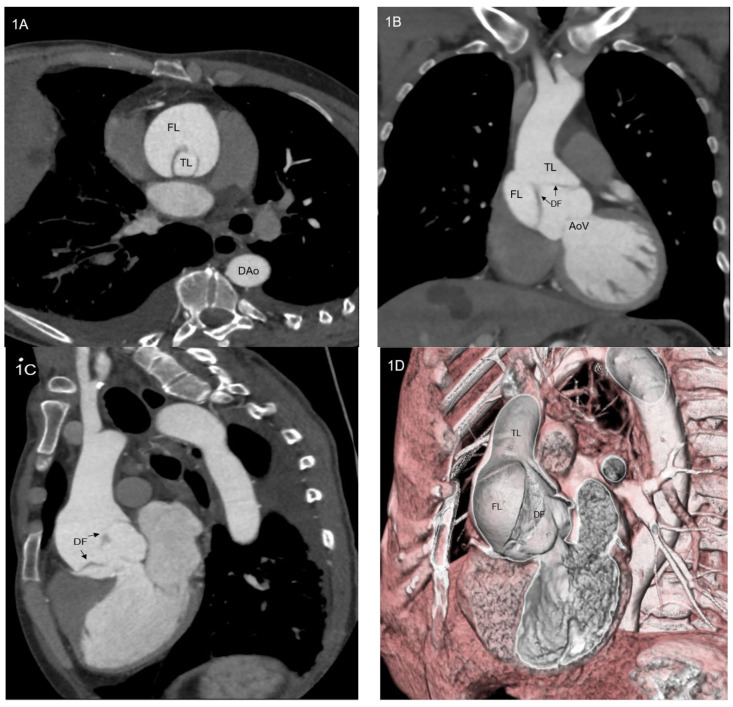
(**A**–**D**) Computed tomography angiogram of a patient with type A aortic dissection: axial scan (**A**), coronal scan (**B**), sagittal scan (**C**) and 3D volume rendering view. AoV = aortic valve; DAo = descending aorta; DF = dissection flap; FL = false lumen; TL = true lumen.

These data could underestimate the real incidence of thoracic aortic dissection, since the hospital-based registries account only for patients who arrived alive at the emergency department, whereas almost 50% of subjects with type A aortic dissection (TAAD, Figure 1A–D) die before hospital admission. Furthermore, a British study suggests that among patients with aortic dissection the proportion of subjects aged over 75 years old will reach 49.7% in 2030 and 57.3% by 2050. This is mainly due to ageing and resistant hypertension [4].

Embryological origin of aorta may have an impact on aortic disease. Ascending aorta and aortic arch derive from neural crest, whereas descending and abdominal aorta derive from paraxial mesoderm [5]. These distinct developmental lineages lead to a different type of smooth muscle cells, expression of matrix metalloproteinase (MMP) and tissue inhibitor metalloproteinase (TIMP); atherosclerosis-related aneurysms are more commonly located in the abdominal aorta and up to 16% of patients with evidence of aortic atherosclerosis develop AAAs. AAA growth rate and rupture can be slowed by smoking cessation, hypertension and plasma-cholesterol levels control [6], although non-atherosclerosis-related risk factors may play a prominent role in ascending aorta and aortic arch aneurysm formation. The mechanical properties of the aneurysmal aorta deteriorate dramatically as the aorta enlarges, reaching critical levels associated with rupture by a diameter of 6 cm. However, a normal aortic size can be present in 35% of cases of TAAD [7].

Nowadays, the first line treatment of acute and chronic ascending aorta diseases is represented by open surgery, but some important innovative devices and techniques have been used for total endovascular surgery of such conditions.

## 2. Aim of the Review

In this paper we first describe first the limitations of open surgery in ascending aorta diseases; subsequently, we discuss in detail the issues related to endovascular approach and the future perspectives of a total endovascular treatment of ascending aorta disease.

## 3. Guidelines Recommendation and Open Surgery Limitations

**Figure 2:**
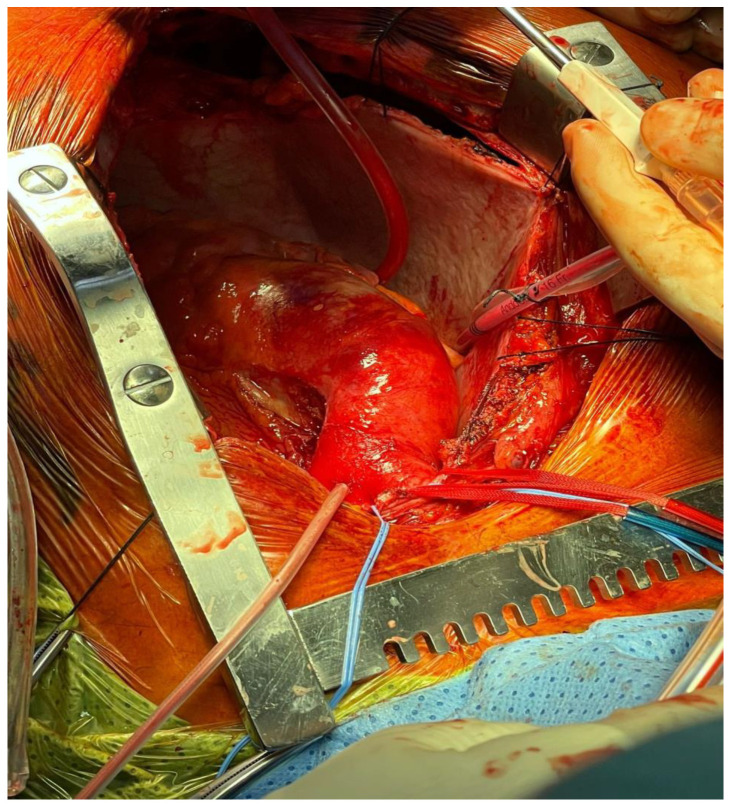
Real picture of type A aortic dissection surgical repair after sternotomy.

Current American and European guidelines recommend urgent surgical intervention (Figure 2) in patients with acute aortic syndrome involving ascending aorta [8,9]. In particular, immediate surgical intervention is recommended in patients with TAAD because of the high risk of life-threatening complications such as aortic rupture and organ malperfusion. The type of surgical intervention can be varied according to the degree of associated aortic valve leaflet pathology, because a valve conduit implantation is necessary only either in case of extensive damage of aortic root or genetic disease. Moreover, extensive arch replacement is recommended only when intimal tear involves the aortic arch or a significant arch aneurysm is observed. In patients with type A intramural hematoma (IMH), urgent surgical aortic repair is indicated only in case of malperfusion, periaortic hematoma, pericardial effusion with cardiac tamponade, refractory pain or aortic rupture. Finally, in patients with penetrating aortic ulcer (PAU), surgery is recommended in case of aortic rupture or associated IMH. However, some patients are deemed too high risk to undergo any surgical procedure, so they are treated with medical therapy alone with consequently poor prognosis [10].

Among patients who have undergone surgical procedure, 30 day mortality is 9.2% overall, 18.3% for emergent dissections or ruptures and 5.5% for aneurysm repair. The long-term survival for 30 day survivors was 86.9%, 77.6%, 52.1%, 38.3% and 26.7% at 5, 10, 20, 30 and 40 years, respectively [11]. Data derived from International registry of Acute Aortic Dissection (IRAD) show that patients with untreated TAAD have the worst prognosis, since their in-hospital mortality is about 57% [12].

A recent observational study [13] of 686 patients with type A aortic dissection found that almost 8% were considered inoperable because of prohibitive or very high surgical risk; prohibitive risk included dementia, advanced malignancy, severe stroke or malperfusion; high risk was attributable mainly to age, frailty, and redo interventions. This study showed that about two-thirds of acute ascending dissection patients who are too high risk for open repair had an anatomy theoretically suitable for stent grafting; notably, the intimal entry tear most often occurred in an area of the aorta that is potentially amenable to coverage with an endovascular device, between the sino-tubular junction (STJ) and innominate artery or distal to the left subclavian artery. Data derived from a Japanese registry showed that 32% of subjects with TAAD were not admitted to surgery and treated with medical therapy; this group had significant increased mortality when compared to patients who underwent surgical intervention (49.7% vs. 11.8%) [10].

In the context of open surgery, some important limitations persist; in the last decade, post-operative 30 day mortality had only a modest decrease from 19.4% to 18.1%. Factors associated with increased 30 day mortality in univariate analysis were older age, operations in the earlier years of the study, presence of diabetes mellitus, preoperative dialysis, hypertension, severe chronic lung disease, immunosuppression, preexisting cerebrovascular disease, concomitant valve procedures, and longer cardiopulmonary bypass time. However, rates of postoperative permanent stroke after repair of AADs remain high (13.8%) and did not improve substantially [14].

Nowadays, considering that a surgical procedure with a 20% of post-operative mortality is not acceptable and the high rate of inoperability, it seems reasonable to offer to these patients an alternative treatment with a total endovascular intervention.

## 4. The Role of Ascending Thoracic Endovascular Aortic Repair

Subjects rejected from open surgery repair may benefit from an endovascular approach. The first stent graft repair of the ascending aorta was reported in 2000 for a chronic ascending dissection [15]. Despite the fact that only case reports, small series and retrospective studies exist [16,17,18], experience with ascending thoracic endovascular aortic repair (aTEVAR) is rapidly evolving, representing an alternative treatment to open repair. Two recent systematic reviews on aortic stenting in patients with TAAD were published [19,20]. In 98% of cases general anesthesia was performed; the most used techniques for blood pressure management were rapid ventricular pacing and medicine-induced hypotension; transesophageal echocardiography was performed ¾ of cases; femoral access was predominant, whereas other vascular approaches were transapical, transaxillary or transcarotid. Procedures were successful in more than 95% of cases. Importantly, 30 day survivals of 91–92.9% and an 80.9% survival after 39 months of follow-up were reported; the most significative complications are represented by new distal dissection, retrograde dissection, stroke and sudden death; stroke occurred in 6% of subjects. Notably, in all these studies the endoprostheses were not projected for ascending aorta stenting but for descending or abdominal district. Therefore, there is a need for dedicated material.

Although these data are encouraging, there are some technical, physiological, and anatomical issues that must be considered.

**Figure 3:**
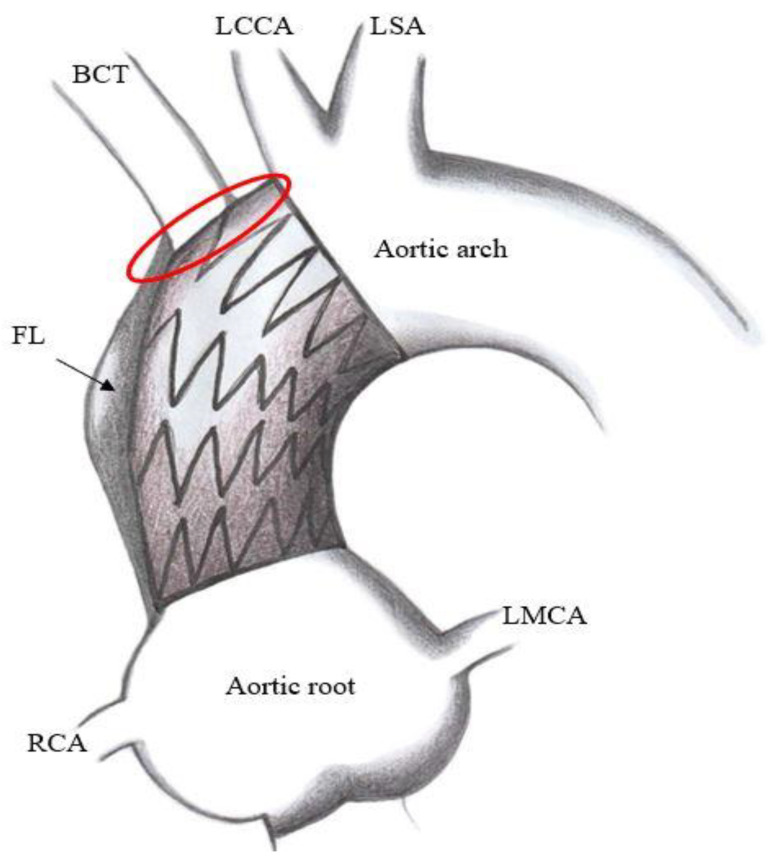
Representation of type A aortic dissection treated with a too long endoprosthesis, covering supra-aortic vessel origins. Red circle indicates supra-aortic vessel covering by the endoprosthesis. BCT = brachiocephalic trunk; FL = false lumen; LCCA = left common carotid artery; LMCA = left main coronary artery; LSA = left subclavian artery; RCA = right coronary artery.

**Figure 4:**
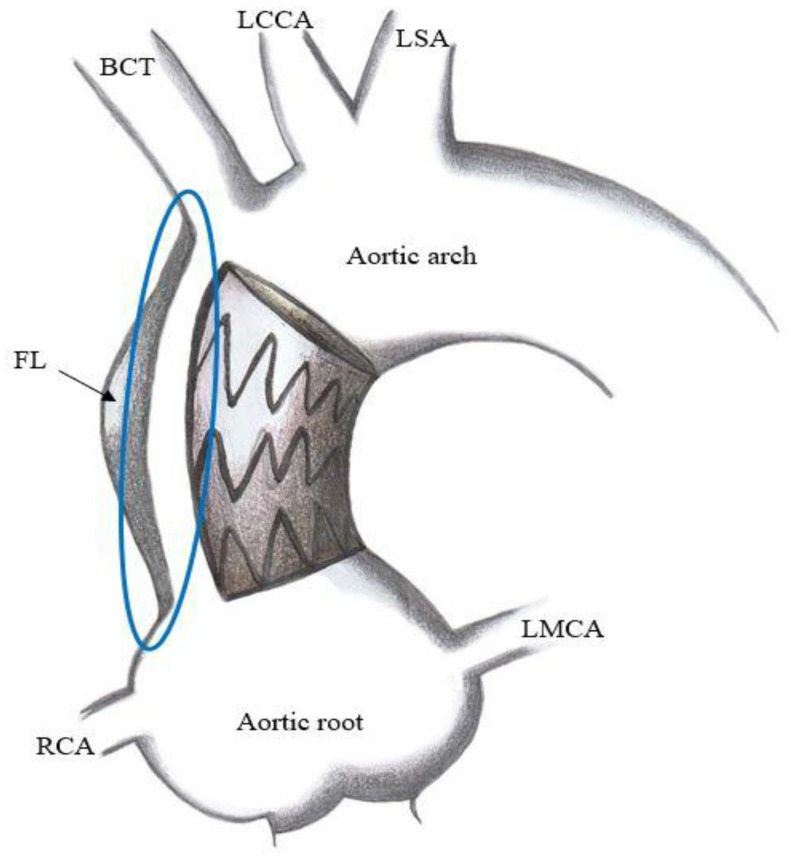
Representation of type A aortic dissection treated with a too short endoprosthesis that does not cover aortic dissection. Blue circle indicate the areas of dissection uncovered by endoprosthesis. BCT = brachiocephalic trunk; FL = false lumen; LCCA = left common carotid artery; LMCA = left main coronary artery; LSA = left subclavian artery; RCA = right coronary artery.

**Figure 5:**
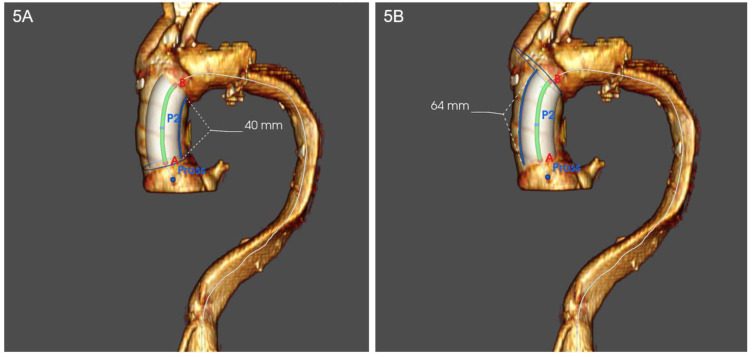
(**A**,**B**) Representation of inner and outer curves of ascending aorta in volume rendering 3D computed tomography angiogram. Inner curve (**A**) appears shorter than outer curve (**B**).

1. Technical issues. Standard thoracic aorta endoprosthesis are too long for ascending aorta stenting (Figure 3) and their nose cones may protrude into left ventricle; their delivery systems do not allow devices manipulation into ascending aorta and across aortic valve and most devices do not account for ascending aorta and aortic arch curves. On the other hand, abdominal aortic devices are too small, and their delivery systems are short for endovascular treatment of ascending aorta (Figure 4). Thus, a trans-subclavian or trans-apical approach was used, even if these procedures are not always feasible.
2. Physiological issues. Ascending aorta presents a complex motion during the cardiac cycle, characterized by a composite of craniocaudal movement and a rotation of 6–14° [21]. Thus, the risk of endoprosthesis dislodgment is not negligible. Although this circumstance could be avoided by device oversizing, this situation can lead to aortic rupture.
3. Anatomical issues. The coverable zone in the ascending aorta is restricted proximally by the aortic sinus with the coronary arteries and distally by the offspring of the brachiocephalic trunk; the most relevant limitation is represented by too proximal entry tears, involving aortic root or aortic valve (<2 cm from the most distal coronary artery); the feasibility of endovascular stenting is not well established and ranges from 2% to 50% of patients with TAAD [22,23,24]; it mainly depends on improper proximal tear sealing, due to the absence of adequate proximal landing zone. Moreover, ascending aorta presents two different curves: a longer outer curve and a shorter inner curve (Figure 5A,B); conventional endoprostheses do not fit this difference in length, so that a proximal shift at inner curve could occur during the endoprosthesis deployment; the proximal shift could provoke impairment of left coronary ostium, leading to serious consequences [25].

## 5. Towards a Total Endovascular Approach to Ascending Aorta: The Endo-Bentall Procedure

Another critical anatomical problem is the involvement of aortic valve with geometrical distortion of aortic annulus which leads to aortic regurgitation (AR).

**Figure 6:**
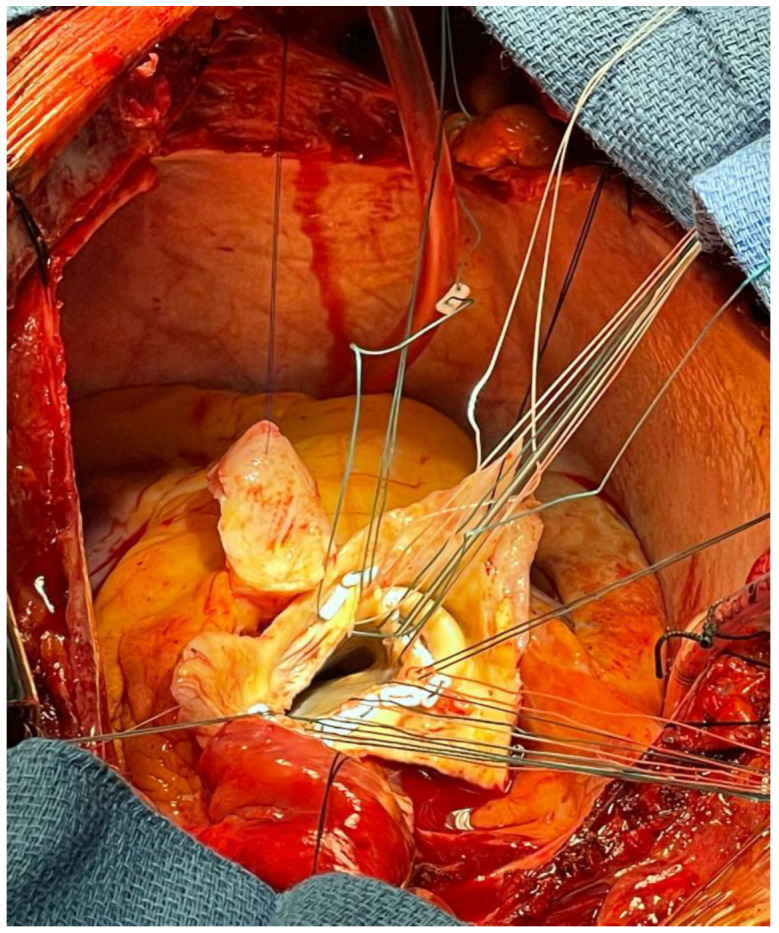
Real picture of Bentall intervention for type A aortic dissection complicated by aortic valve regurgitation, showing ascending aorta resection and aortic valve exposure.

AR complicates TAAD in 40–75% of cases [9]. In type I AR associated with ATAD, there is acute dilation at level of STJ (type Ia), of the aortic root (type Ib), or of aorto-ventricular junction (type Ic). An excessive cusp mobility can be observed in type II AR because of symmetric or asymmetric detachment of the commissural attachments [26]. When the aortic valve is dramatically involved, simultaneous aortic repair and aortic valve replacement is mandatory (Figure 6). Combining transcatheter aortic valve implantation (TAVI) with TEVAR into one device, in the so-called “endo-Bentall” procedure, would mean to proximally deploy the endograft in an undissected stable area, with a secondary potential sealing zones at the STJ level and at the level of the brachiocephalic trunk [27].

Rylski at al. [28] used a different device consisting of a transapically implantable endovascular valved conduit, with a proximal transcatheter aortic valve connected to an uncovered portion of a covered stent graft; this device sealed the entry tear in the ascending aorta, ensuring coronary perfusion and treating aortic regurgitation; moreover, any pericardial effusion can be drained via transapical approach. Its sealing power is considerable since it has three different landing zones, located in the aortic valve annulus, at the level of the STJ, and at distal ascending aorta.

**Figure 7:**
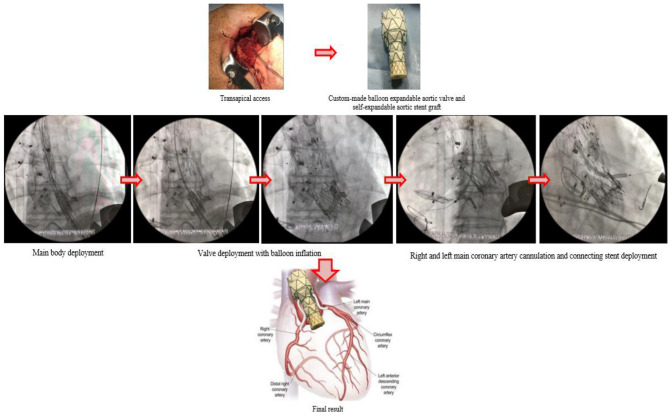
Figure reporting the step-by-step procedure published by Gaia in 2020. First, a femoral arterial access for angiography and femoral venous access for pacing are obtained; RCA and LMCA are cannulated; then a transapical access with a 30 Fr introducer is obtained; consequently, endoprosthesis is positioned and deployed; after that, balloon expandable prosthetic aortic valve is deployed. Then, RCA and LMCA are cannulated through endoprosthesis main body and coronary branches and connecting stents are finally deployed. Courtesy of Professor Diego Gaia, Federal University of Saõ Paulo, Brazil. LMCA = left main coronary artery; RCA = right coronary artery.

Gaia et al. [29] first introduced a custom-made device for the endo-Bentall procedure; it consisted of a proximal portion, with a balloon-expandable transcatheter aortic valve prosthesis, and a distal portion, with a self-expandable aortic stent graft; moreover, there are two small branches in the proximal portion for coronary artery catheterization and bridge with covered stent-grafts (Figure 7).

Gandet in 2021 used a modified endograft with three fenestration for coronary ostia blood flow in ascending aorta, followed by TAVI; this procedure was performed using a transapical and transfemoral “rendez-vous access” [30]. However, this technique does not warrant a secure seal at the level of coronary arteries. Table 1 summarizes available technical aspects of endo-Bentall procedure.

Although endo-Bentall intervention is an attractive novelty, some points must be discussed. A device implanted in ascending aorta is subject to different movements and torsions in respect to endoprosthesis of thoraco-abdominal aorta; furthermore, the risk of bridge coronary stents fracture, dislocation and thrombosis is not negligible, therefore dedicated coronary stents are needed. Conventional devices for TAVI are designed for calcified valves that provide a structural support; in case of AR alone, a considerable risk of device migration exists. Finally, some patients may have coverage of patent coronary artery vein graft during ascending aorta stenting.

## 6. Clinical Trials

Results from the ARISE early feasibility study were recently published (First-In-Human Evaluation of a Novel Stent Graft to Treat Ascending Aortic Dissection) [31]. This is a prospective, multicenter, non-randomized, single-arm study to assess the feasibility of the treatment of DeBakey type I/II aortic dissections. The primary endpoint is represented by post-procedural 30 day all-cause mortality. The study enrolled 19 patients and 100 patients were excluded. A single device, or multiple devices in an overlapping manner, may be used. This stent graft’s unique feature is the ability to partially deploy, and then the inner curve can be shortened to allow orthogonal alignment with the sinotubular junction (STJ) and to match the curve of the aorta. Device selection is based on the overall diameter of the aorta at the STJ, proximal and distal ascending landing zone; since the length of the ascending aorta outer curve is superior to the inner curve, an oversizing of 6–33% is recommended. The primary endpoint occurred in 3/19 (15.8%). Causes of death included ischemic cardiomyopathy, severe aortic valve insufficiency, and aortic rupture. No deaths beyond 30 days were observed, but the composite endpoint of all-cause mortality, myocardial infarction, or major stroke occurred in 23.8% of subjects. All strokes with one exception occurred within the first week post-operatively. Some procedural complications included one case of development of severe aortic insufficiency requiring valve repair and ascending replacement; one case of right ventricular perforation from the temporary pacemaker lead, treated with urgent cardiac surgery in one patient; finally, one patient had type Ic endoleaks with no resolution. No cases of ASG device migration were reported.

Another interesting clinical trial is the EVOLVE Aorta (Endovascular Treatment of Thoracic Aortic Disease) [31]. This is a non-randomized trial that aims to assess the role of endovascular therapy in three different arms: first, reparation of aneurysm or dissection of ascending aorta; second, reparation of aneurysm or dissection of aortic arch; third, thoracoabdominal aortic disease. Primary outcomes are all-cause mortality, stroke and/or transient ischemic attack (TIA), aneurysm-related death. The enrollment started on October 2018, and it will be completed in 2027 with an estimated number of 170 participants.

## 7. Conclusions

Acute and chronic ascending aorta dissections lead to poor prognosis if treated with medical therapy only, but a consistent portion of subjects is often excluded from open repair surgery. The endovascular treatment of ascending aorta needs different devices from those used in descending and abdominal aorta for some important technical, physiological, and anatomical reasons. Along with the introduction and development of new models of endoprosthesis and delivery systems, encouraging data from case series and early feasibility study have been reported in literature. The experience in this field is rapidly evolving but more robust data are expected from ongoing clinical trials to better understand the clinical impact of this innovative approach.

## Figures and Tables

**Table 1:** Summary of Literature of endo-Bentall techniques and materials.

Author, Publication Year	Patient’s Age	Gender	Surgical Risk	Aortic Disease	Vascular Access	Diseased Aortic Valve	Endoprosthesis and Valves	Coronary Arteries Perfusion
Rylski, 2014 [28]	-	-	-	Ascending aorta aneurysm	Transapical	Native aortic valve stenosis	Endovascular valvuled conduit: proximal transcatheter aortic valve connected to an uncovered portion of a covered stent graft	Stented endoprosthesis
Gaia, 2020 [29]	64	female	EuroSCORE 25.8%	ascending aorta pseudoaneurysm	Transapical	Biological prosthetic valve failure	Custom-made balloon expandable aortic valve and self-expandable aortic stent graft	Coronary branch from the endoprosthesis
Gandet, 2021 [30]	82	female	-	Ascending aortica aneurysm	Transapical and transfemoral (“Rendez-vous-access”)	Native aortic valve stenosis	Fenestrated physician-modified endograft	Endoprosthesis fnestration

## Data Availability

Data will be available on demand from the corresponding author.

## Abbreviations

AAA	abdominal aortic aneurysm
aTEVAR	ascending thoracic endovascular aortic repair
MMP	matrix metalloproteinase
TAA	thoracic aortic aneurysm
TAAD	type A aortic dissection
TAD	thoracic aortic dissection
TIA	transient ischemic attack
TIMP	tissue inhibitor metalloproteinase
